# Robust Approach for Nonuniformity Correction in Infrared Focal Plane Array

**DOI:** 10.3390/s16111890

**Published:** 2016-11-10

**Authors:** Ayoub Boutemedjet, Chenwei Deng, Baojun Zhao

**Affiliations:** School of Information and Electronics, Beijing Institute of Technology, Beijing 100081, China; bout_ayoub@yahoo.com (A.B.); zbj@bit.edu.cn (B.Z.)

**Keywords:** nonuniformity, correction, scene-based, bad pixels

## Abstract

In this paper, we propose a new scene-based nonuniformity correction technique for infrared focal plane arrays. Our work is based on the use of two well-known scene-based methods, namely, adaptive and interframe registration-based exploiting pure translation motion model between frames. The two approaches have their benefits and drawbacks, which make them extremely effective in certain conditions and not adapted for others. Following on that, we developed a method robust to various conditions, which may slow or affect the correction process by elaborating a decision criterion that adapts the process to the most effective technique to ensure fast and reliable correction. In addition to that, problems such as bad pixels and ghosting artifacts are also dealt with to enhance the overall quality of the correction. The performance of the proposed technique is investigated and compared to the two state-of-the-art techniques cited above.

## 1. Introduction

Focal Plane Array (FPA) sensors technology have undergone significant maturation since the beginning of their development in the 1970s in the last century. They brought an enormous contribution to different imaging systems, especially in the field of infrared. Infrared FPA (IRFPA) sensors are known for their wide range of applications in different domains such military, medical imaging, surveillance and instrumentation. However, they have some disadvantages affecting the image quality. The most important downside is the spatial photo response nonuniformity, which arises because each individual detector element in the array exhibits a response characteristic differing from those of its neighboring elements. Therefore, it produces an undesirable “fixed pattern noise” (FPN) that is superimposed on the image obtained from the device [1]. Additionally, the main challenging part of the nonuniformity is the slow drifting of the spatial nonuniformity in time. Thus, a one-time factory calibration will not provide a permanent solution to the problem that makes reference-based nonuniformity correction (NUC) using uniform blackbody irradiance sources not suitable for continuous long time applications. Hence, scene-based NUC (SBNUC) methods were introduced to assure continuous correction during normal functioning of the camera.

One category of SBNUC techniques is based on spatiotemporal assumptions on some scene statistics, which will be used to estimate the correction needed for the array [2,3,4,5]. These statistical approaches are adopted for their lower computational complexity and better real-time performance. However, they require a relatively big number of image frames to satisfy their premade assumptions.

Another SBNUC method is the registration-based approach, which exploits the global motion between frames by considering that each detector in the array should respond identically when observing the same scene point [6,7,8,9,10,11,12,13]. Considering a pure translation motion model for interframe shifts, Ratliff et al. [7,8] developed a process that compensates for bias nonuniformity, while Zuo et al. [9,10,11] provided both gain and bias correction methods. Black et al. [12,13], on the other hand, offered an approach to deal with both high- and low-spatial frequency nonuniformity. Despite the registration-based technique being highly effective compared to other methods, it relies mainly on an accurate registration. This leads us to its main challenge that is assuring reliable registration in practical applications conditions like: highly corrupted frames, the presence of local motion in the scene independent from the global one, and complex motion features between frames rather than pure horizontal and vertical motions. Recently, a lot of research has focused on facing these challenges, and the most relevant to our work is the one conducted by Zuo et al. [10] where the authors proposed three strategies to overcome the problems cited above, and these strategies will be discussed in detail in the next section.

Another class of SBNUC methods is the adaptive NUC approach, which uses a least mean square (LMS) algorithm to adaptively determine the NUC parameters based on a “target” image that is formed using a spatial low pass filter on the observed frame [14,15,16,17]. This technique has a good ability for noise reduction, but it suffers from ghosting artifacts mainly near the edges where they burn in the background due to a smoothing effect.

Our method is a combination of the last two discussed SBNUC techniques, and it exploits their strongest assets along with avoiding their issues, in addition to more support strategies to enhance the overall quality of the corrected images. We also compare our results with two novel methods from both SBNUC techniques, namely: interframe registration-based NUC (IRNUC) [10] and adaptive SBNUC based on neural network (SBNUC-NN) [17].

This paper is organized as follows. Section 2 is dedicated to a detailed description of the proposed algorithm along with highlighting the improvements made. Then, we present the simulation results in Section 3 followed by a brief discussion in Section 4 and wrap up with conclusions in Section 5 that sum up the presented work.

## 2. Method Description

### 2.1. The Correction Process

Ideally, the correction process should maintain, under any circumstances, its correction ability while the FPN is still present to ensure that we propose a method based on the use of two known NUC processes so we can enlarge the range of operational conditions where our process could work effectively. This method is explained in detail in this section.

Although the nature of nonuniformity in an FPA is nonlinear, many works state that it can be approximated by a linear model, especially for a short time span when the irradiance does not change significantly. A conventional linear model was commonly adopted to represent the output of an individual detector as a linear function of the received irradiance in the following way:

(1) $$y_{n}\left(i,j\right)=a_{n}\left(i,j\right)x_{n}\left(i,j\right)+b_{n}\left(i,j\right),$$

where $y_{n}\left(i,j\right)$ is the observed irradiance at the ${\left(i,j\right)}^{th}$ detector, $a_{n}\left(i,j\right)$ and $b_{n}\left(i,j\right)$ are the respective gain and bias of the detector, $x_{n}\left(i,j\right)$ accounts for the real irradiance collected by this detector and *n* is the frame index.

The NUC process is a linear mapping of the observed irradiance that estimates the true irradiance using NUC coefficients as follows:

(2) $${\hat{x}}_{n}\left(i,j\right)=w_{n}\left(i,j\right)y_{n}\left(i,j\right)+v_{n}\left(i,j\right),$$

where $w_{n}\left(i,j\right)$ and $v_{n}\left(i,j\right)$ are the NUC gain and bias, respectively, which are related to the nonuniformity gain and bias by the following relations: $w_{n}\left(i,j\right)=\frac{1}{a_{n}\left(i,j\right)}$ and $v_{n}\left(i,j\right)=-\frac{b_{n}\left(i,j\right)}{a_{n}\left(i,j\right)}$.

Inspired from the neural network architecture, the determination of the NUC coefficients is conducted by minimizing the mean square error between the estimation of the current frame and a target image $T_{n}$ using the steepest descent method. The error used by this method is given by:

(3) $$e_{n}\left(i,j\right)={\hat{x}}_{n}\left(i,j\right)-T_{n}\left(i,j\right),$$

where ${\hat{x}}_{n}\left(i,j\right)$ is calculated by Equation (2) using the last updated NUC coefficients.

In the case of adaptive NUC, the target image is a filtered version of the observed image using, for example, a mean filter [16] or a Gaussian filter [17]. In the other case, the target image of the registration based NUC is a properly shifted version of a previously corrected frame.

The choice of using one of these two target images is made according to the level of noise. The filtered version offers a good smoothing of the image, which will reduce significantly the level of noise, but it suffers from edge smearing where they are burnt in the background. While the registered version gives a better estimation of the current frame with preservation of the image details, however, its good performance relies completely on a correct estimation of the shifts between the two frames, which is strongly affected by noise.

Based on the above discussion, we propose a way to use both techniques in a complementary way depending on the level of noise present in the frame. To do so, a decision criterion is established comparing the noise level in the current frame to a threshold value. If it is bigger, we use the filtered version as a target image to get the smoothing effect. Otherwise, we use the shifted one as the level of noise will not trouble the registration process.

Since we do not have access to the clear image, the noise level is estimated by computing the mean square error between the current observed frame corrected with the last updated coefficients, and a smoothed version of it using a 3 × 3 mean filter. The calculated error is formulated as:

(4) $$E=\frac{1}{N.M}\sum_{i=1}^{N}\sum_{j=1}^{M}{\left({\hat{x}}_{n}\left(i,j\right)-{\hat{x}}_{n}^{*}\left(i,j\right)\right)}^{2},$$

where ${\hat{x}}_{n}^{*}\left(i,j\right)=\frac{1}{9}\sum_{k=-1}^{1}\sum_{l=-1}^{1}{\hat{x}}_{n}\left(i+k,j+l\right)$ is the filtered version of the frame ${\hat{x}}_{n}$, and *N* and *M* represent the size of the frame.

**Figure 1:**
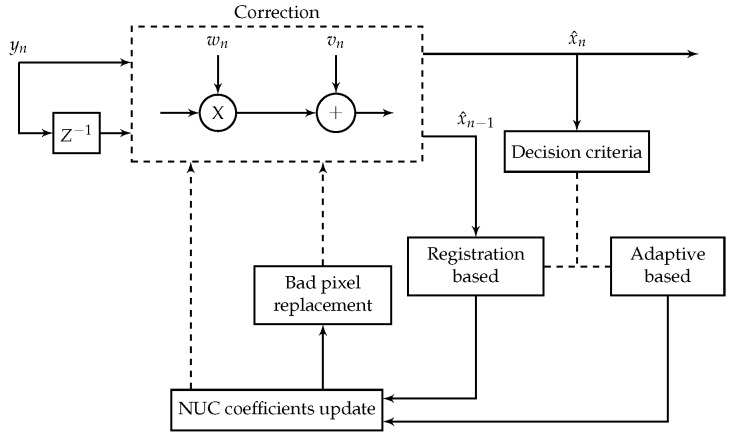
Scheme of the proposed method.

Finally, the corresponding error $e_{n}$ is injected into the following NUC coefficient update process based on the steepest descent method:

(5) $$\left\{\begin{array}{ccc} w_{n}\left(i,j\right) & = & w_{n-1}\left(i,j\right)-\alpha_{n}e_{n}\left(i,j\right)y_{n}\left(i,j\right),\\ v_{n}\left(i,j\right) & = & v_{n-1}\left(i,j\right)-\alpha_{n}e_{n}\left(i,j\right), \end{array}\right.$$

where $\alpha_{n}$ is the convergence step that controls the speed of convergence of the process. If it is set to a high value, it assures a fast convergence, and if it is set to a low value it provides good stability. A complete scheme representing the whole correction process is presented in Figure 1.

### 2.2. Registration Algorithm

Since the motion model considered is a pure translation, we studied methods proposed in the literature that only compute the linear horizontal and vertical shifts between two images. One of these classic algorithms is based on the computation of the cross correlation function between the two images given by:

(6) $$c\left(x,y\right)=\left|F^{-1}\left(C\left(u,v\right)\right)\right|=\left|F^{-1}\left(\frac{F\left(r\right)F^{*}\left(y\right)}{\left|F\left(r\right)F^{*}\left(y\right)\right|}\right)\right|,$$

where F(.) represents the Fourier transform, $F^{*}\left(.\right)$ its complex conjugate and $F^{-1}\left(.\right)$ its inverse transform, *r* and *y* are the reference and current image, respectively. The position of the peak of c(x,y) in the horizontal and vertical axes will represent the horizontal and vertical displacement $\delta_{x}$ and $\delta_{y}$, respectively. For an accurate registration with a subpixel precision, the cross-correlation peak is upsampled using matrix-multiply discrete Fourier transform (DFT) [18].

In the presence of FPN, however, the performance of the above technique is reduced due to the way the noise is presented in the cross correlation, especially around the origin point, which may lure the process of locating the peak into error. Zuo et al. [10] proposed some strategies to overcome this problem, for instance: masking the origin and its neighboring pixels in c(x,y) with zeros, and stopping the update of the correction coefficients when the displacement or the peak amplitude are not sufficient. These techniques provide good results, especially in the case of a continuously moving camera, but in the situation where the movement of the camera is halted or not sufficient for a considerable amount of time, the update of the NUC parameters is stopped, which slows down the correction process.

In the proposed method, we keep the same classic registration algorithm and try to reduce the FPN to an acceptable level. This way, we assure the detection of small displacement and avoid the need of ignoring them during the correction process due to the masking effect.

### 2.3. Anti-Ghosting Measures

First, we discuss the convergence rate value, and here we differ between the two cases of the correction method used. For the adaptive NUC, it suffices to set the convergence step to a properly chosen fixed value that produces a good smoothing effect during just a few frames.

In the case of registration-based NUC, we know the error $e_{n}$ will be computed over the overlapping area between the two adjacent frames only, and it will represent ideally the NU left in the current frame. However, in real situations, this error will also be affected by other factors that may corrupt the correction results.

One of these factors is local motion, which stands for presence of features in the image with different motion models from the global one. Zuo et al. [10] proposed an efficient strategy to deal with this problem that we also adopted for our method. They used a simple rule to exclude the abnormal data that comes from local motion or bad pixels. The rule is based on the assumption that the nonuniformity follows a Gaussian distribution with a mean $\mu_{n}$ and a standard deviation $\sigma_{n}$ where any error value that deviate from this distribution will be excluded as follows:

(7) $${\overset{´}{e}}_{n}\left(i,j\right)=\left\{\begin{array}{ccc} 0 & , & \left|e_{n}\left(i,j\right)-\mu_{n}\right|\geq 3\sigma_{n},\\ e_{n}\left(i,j\right) & , & \left|e_{n}\left(i,j\right)-\mu_{n}\right|<3\sigma_{n}, \end{array}\right.$$

where $\mu_{n}$ and $\sigma_{n}$ are calculated over $e_{n}$. The new error ${\overset{´}{e}}_{n}$ will be the one injected in the correction process in Equation (5).

Another important factor affecting the correction is complex motion, which means that in real conditions the global motion will not always be purely horizontal or vertical displacement. Other motion models can be present as well. In this case, an additional error due to inaccurate registration will be present. To address this problem, we adopted an adaptive convergence step. The step size $\alpha_{n}$ (see Equation (5)) decreases in the case of complex motion and increases when an accurate registration is performed. Detailed analysis will be given in Section 3.2.

### 2.4. Bad Pixel Replacement

As it was mentioned above, bad pixels are excluded from the error, along with the abnormal data, and will not be corrected. Thus, it is necessary to find an alternative to deal with them, but first we need to detect them in the scene.

As we know, bad pixels are characterized with an abnormal value compared to their neighboring pixels, inspired by that and the sigma filter [19], we use the following process to correct them: let $x_{n}\left(i,j\right)$ be the inspected pixel and $\Phi =\{x_{n}^{k}\}$ its 3x3 neighboring pixels, then:

(8) $$K\left(i,j\right)=\sum_{k\in \Phi }\delta_{k},\;where:\delta_{k}=\left\{\begin{array}{ccc} 1 & & if\;\left|x_{n}^{k}-x_{n}\right|>\Delta ,\\ 0 & & if\;\left|x_{n}^{k}-x_{n}\right|\leq \Delta , \end{array}\right.$$

where K(i,j) represents the number of pixels with values quite different from the central pixel value. This number is then compared to a threshold $K_{s}$. If it is bigger, the pixel value $x_{n}\left(i,j\right)$ is considered abnormal, and it will be replaced by the median value of its neighboring pixels:

(9) $${\overset{´}{x}}_{n}\left(i,j\right)=\left\{\begin{array}{ccc} median(x_{n}\left(i,j\right)) & & if\;K\left(i,j\right)>K_{s}.\\ x_{n}\left(i,j\right) & & if\;K\left(i,j\right)\leq K_{s}. \end{array}\right.$$

Parameters Δ and $K_{s}$ are essential in this process. First, Δ is used to detect pixels with values deviating from the central pixel value, in other words, pixels that do not belong to the same region as the central pixel. Therefore, if Δ is set too high, the ability to detect these pixels will decrease, which increases the probability of wrongly considering the central pixel normal. Otherwise, if it is set too small, more pixels will be added to K(i,j) and the probability of wrongly considering the central pixel abnormal will increase. Second, $K_{s}$ represents the maximum number of pixels with intensities different from the intensity of the central pixel where the latter is considered a normal pixel. This parameter will allow us to make the difference between a pixel that belongs to an edge and an abnormal pixel.

**Figure 2:**
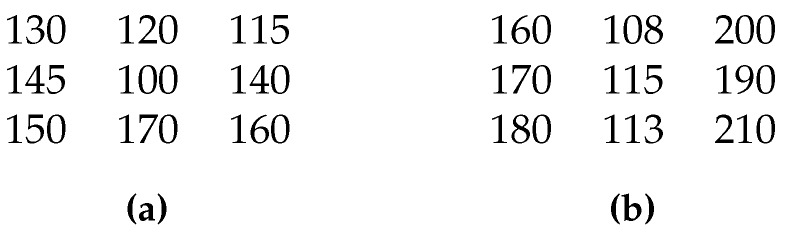
Comparison examples for the bad pixel replacement process (**a**) case of an abnormal pixel (**b**) case of a pixel that belongs to an edge.

A comparison example is also shown, in simplified form, in Figure 2 to further explain the role of these parameters. The first example depicts the case of an abnormal pixel where we can see that the central pixel value is quite different from other pixels values. The second one shows the case of a pixel that belongs to an edge where some neighboring pixels have values near the value of the central pixel, which means that they belong to the same region in the scene.

We set $K_{s}=7$ and Δ=10 for both examples. The first one provides K(i,j)=8 and successfully detects the central pixel as abnormal. The second one gives K(i,j)=6, which means that the central pixel is normal. Now, if we set Δ=5 in the second example, K(i,j) will become 8 and the central pixel will be wrongly replaced. In addition, if we set Δ=20 in the first example, K(i,j) will become 6 and the central abnormal pixel will not be replaced.

We found during experiments that for 3 × 3 neighboring pixels, $K_{s}=7$ and Δ=10 are suitable values to ensure the good functioning of this process.

## 3. Results

The aim of this part is to test the efficiency of our method by analyzing its correction performance. Consequently, a comparison with existing methods is made to point out and validate the main improvements. The metric used for performance evaluation is the PSNR, which stands for the peak signal-to-noise ratio, and it is defined as follows:

(10) $$PSNR=20\times log_{10}\left(\frac{2^{b}-1}{RMSE}\right),$$

where *b* is the number of bits that represents a pixel value in the image (8 bits in our case), RMSE is the root mean square error between the estimated image ${\hat{x}}_{n}$ and the clear one $x_{n}$, and it is computed as:

(11) $$RMSE=\sqrt{\frac{1}{N.M}\sum_{i=1}^{N}\sum_{j=1}^{M}{\left({\hat{x}}_{n}\left(i,j\right)-x_{n}\left(i,j\right)\right)}^{2}},$$

where *N* and *M* are the image dimension.

All sequences of grey scale images used here are obtained from the dataset related to the work done by Portmann et al. [20].

### 3.1. Registration Accuracy

**Figure 3:**
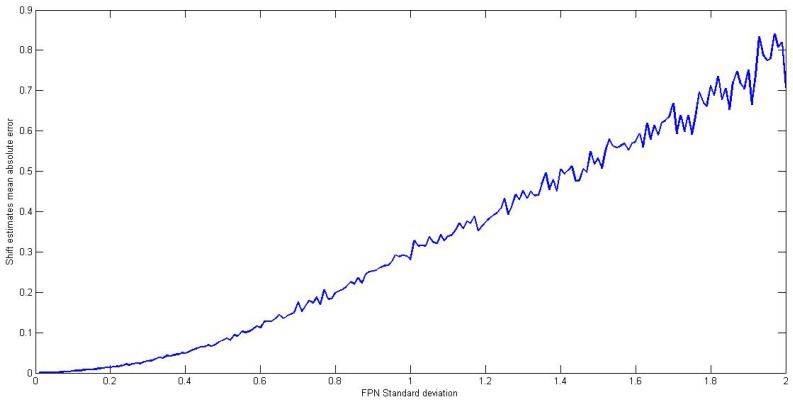
Mean absolute error in shift estimates with regard to the bias nonuniformity standard deviation.

First, we performed a study on the sensibility of the registration algorithm to different levels of noise. We know that the accuracy of this algorithm is strongly deteriorated in the presence of an increased amount of FPN. To demonstrate that, we ran the algorithm on an image sequence with known shifts and for different levels of noise. The mean absolute error in the shift estimates is then computed and represented as a function of the noise standard deviation, and the result is shown in Figure 3. The noise was generated using a zero-mean Gaussian model for bias nonuniformity with 50 as standard deviation, and the image sequence is a 200 frame, 324 × 256, 8-bit images.

It can be noticed that the error in shift estimates increases as the level of nonuniformity increases. Based on the obtained results, and according to the desired precision we want to achieve with the registration algorithm, we can extract the maximum level of noise allowing a shift estimates error less than this precision, which will be our threshold to switch between adaptive and registration-based as the correction method.

### 3.2. Parameters and Experimental Setting

The convergence step $\alpha_{n}$ introduced in Equation (5) plays an important role in the convergence and stability of the correction. Higher value provides fast convergence but may cause some instabilities, while smaller value ensures a stable but slow correction. In our method, we distinguish the two cases of the correction used: adaptive or registration-based.

For the adaptive correction, the convergence step can take higher value to produce fast correction with good amount of noise reduction in few frames before switching to the registration-based correction. Thus, a fixed value of 0.5 was used during the adaptive correction phase in our method. However, in the case of adaptive correction only, this high value will engender ghosting artifacts especially around edges and during lack of motion of the camera, so an adaptive convergence step should be used [17]. Hence, for the SBNUC-NN algorithm used for comparison with our method, we implemented an adaptive convergence step proportional to the amount of noise left in the image: $\alpha_{n}=C\times E$, where *E* is defined in Equation (4). *C* is a scaled constant that assures values of $\alpha_{n}$ not exceeding 0.2 (in our experiments, we set C=5).

For the registration-based correction, as we mentioned in the previous section, $\alpha_{n}$ is adaptively set to reduce the effect of non-translation motion on the correction. This can be achieved by introducing a ratio between the maximum amount of noise expected that corresponds to NU and the amount of noise present in the frame. Therefore, the convergence step is set as follows:

(12) $$\alpha_{n}=\alpha_{max}\times \frac{E_{max}}{E},\;E=\frac{1}{P.Q}\sum_{i=1}^{P}\sum_{j=1}^{Q}{\left({\overset{´}{e}}_{n}\left(i,j\right)\right)}^{2},$$

where $\alpha_{max}$ stands for the maximum value of the convergence step (we use the value 0.05 recommended in [10]), $E_{max}$ represents the maximum error associated to the NU and *E* is the mean square error over the overlapping area with dimension *P* × *Q*. In the presence of complex motion and since the registration process considers only pure translation, a registration error appears and adds to the NU already present, which makes the error *E* bigger than $E_{max}$. Therefore, the value of $\alpha_{n}$ will decrease to reduce the effect on correction parameters. In our work, $E_{max}$ is set to the value of *E* in Equation (4) in the last frame before the process switched to the registration-based correction.

**Figure 4:**
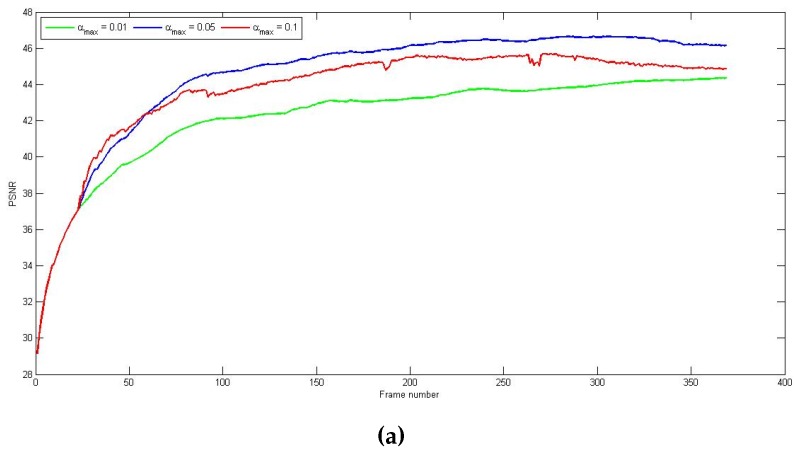

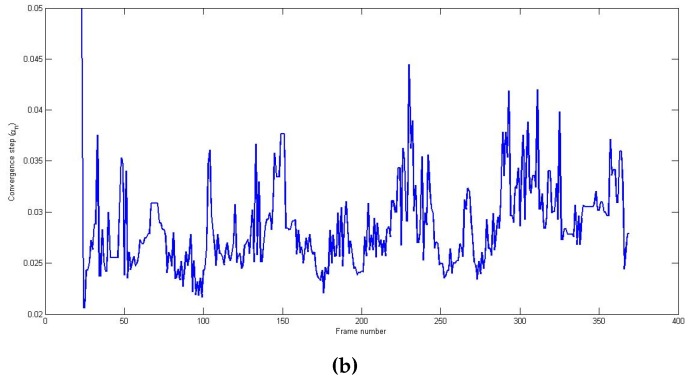
(**a**) Peak signal-to-noise ratio (PSNR) as a function of the frame number for different values of $\alpha_{max}$ (**b**) convergence step $\alpha_{n}$ of the registration-based correction.

The effect of these parameters is shown in Figure 4. Note that the algorithm switched to registration-based correction at the 23rd frame. In Figure 4a, we have the resulting PSNR from three different $\alpha_{max}$ used, and we can see that a bigger value boosts the convergence speed at the beginning, but, eventually, instabilities will cause the correction performance to decrease. On the other hand, a smaller value offers a good stability but the correction converges slowly. Hence, an optimal value is chosen ($\alpha_{max}=0.05$), and the resulting convergence step is depicted in Figure 4b.

As for the IRNUC algorithm used for comparison, we adopted the convergence step recommended in [10] given by $\alpha_{n}=\alpha_{max}\times C_{max}$, where $C_{max}$ is the peak of c(x,y) (see Equation 6) corresponding to the shift. Note that, for the first 50 updates, Zuo et al. fixed the convergence step to its maximum value $\alpha_{max}=0.05$ to avoid noise effect on it.

### 3.3. Correction Efficiency

The nonuniformity was simulated using a zero mean Gaussian model with 50 as the standard deviation for the bias and a unit-mean Gaussian model with 1 as the standard deviation for the gain. The first sequence (referred here as sequence 1) contains 370 frame, 324 × 256, 8-bit images.

Our simulation focused on the comparison between the proposed algorithm and two state-of-the-art NUC techniques, namely SBNUC-NN [17] and IRNUC [10].

**Figure 5:**
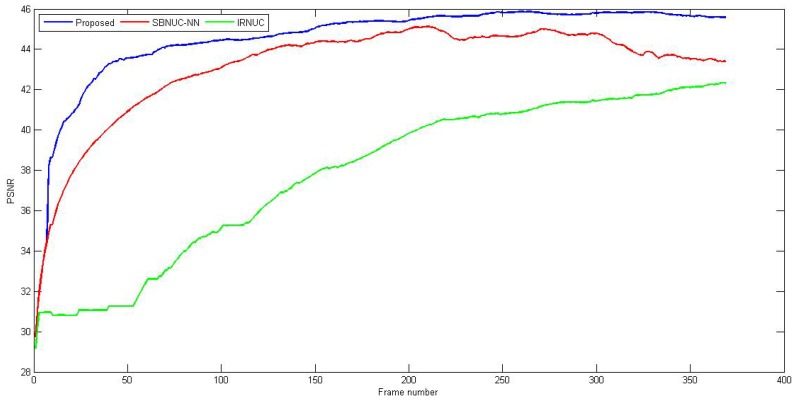
PSNR as a function of the frame number of sequence 1 for the three correction algorithms: proposed (**Blue**), adaptive (**Red**), registration-based (**Green**).

**Figure 6:**
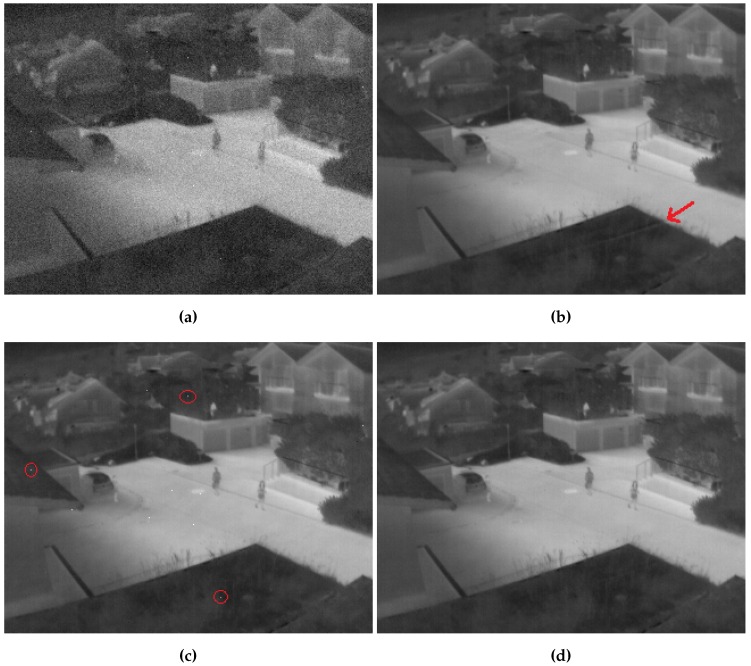
Frame 250 of sequence 1 (**a**) with simulated nonuniformity; (**b**) corrected with the adaptive algorithm; (**c**) corrected with registration-based algorithm; and (**d**) corrected with proposed algorithm.

Results for sequence 1 are represented in Figure 5 and Figure 6. As we can see in the PSNR graph, the proposed method demonstrates rapid convergence with good stability during operation and better performance than other methods. With a shifting threshold that equals 0.0001 and a 0.05 maximum convergence step, it needed few frames to shift to registration-based correction and a few more few dozen to converge to its maximum correction. SBNUC-NN started with good performance along with the proposed algorithm, but, eventually, its performance decreases when the camera does not move sufficiently, due to ghosting artifacts that start to appear around the edges, as we can see in Figure 6b. IRNUC, on the other hand, converges slowly due to a relatively big amount of noise and complex camera motion. Its performance then starts to increase and reach a level close to that of the proposed method, but it still suffers from bad pixels clearly appearing in Figure 6c.

Both nonuniform and corrected sequences are represented in video 1 and video 2 respectively.

**Figure 7:**
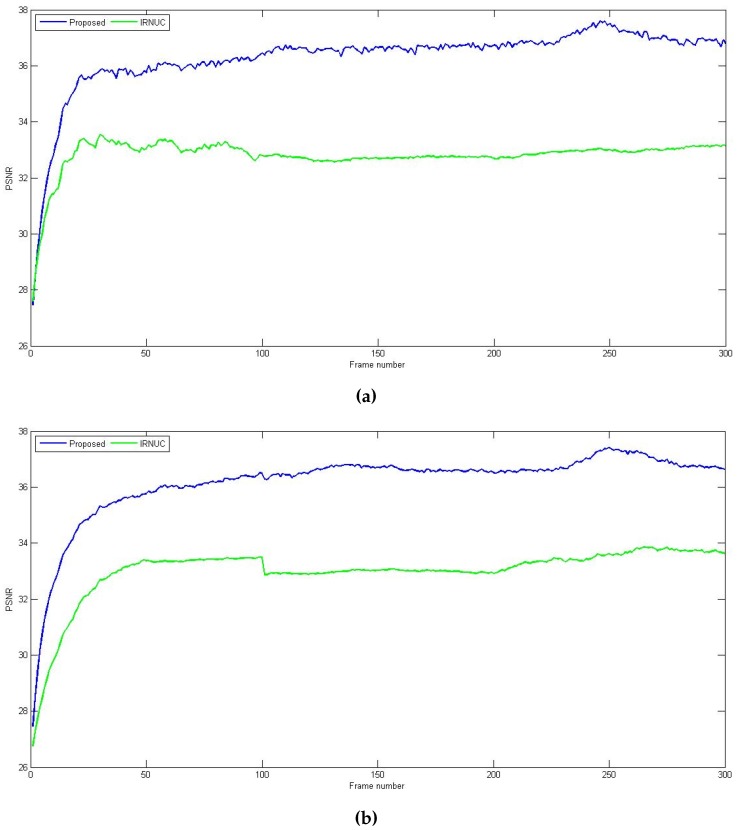
PSNR as a function of the frame number of sequence 2 for the two correction algorithms: Proposed (**Blue**), registration-based (**Green**) (**a**) with the same FPN (**b**) with a simulated drift in the FPN.

The second sequence (referred to here as sequence 2) contains 300 frame, 256 × 256, 8-bit images, and this sequence is used to simulate the lack of motion effect on the correction process. To do so, the frames were generated as follows: the first 100 frames exhibit horizontal and vertical shifts greater than 2, the next 100 frames have shifts less than 1 pixel and the last 100 frames have again shifts greater than 2 pixels. First, we use the same simulated FPN for all the frames, and the resulting PSNR for the proposed and IRNUC algorithms are represented in Figure 7a.

In addition to the fact that the proposed method achieves faster and greater amounts of correction, we can clearly see that, for the set of frames where the shifts are small (between 100 and 200), the IRNUC based algorithm keeps the same performance. This occurs due to the correction parameters update process being halted as a result of lack of motion. Meanwhile, the PSNR of the proposed method continues to rise despite this fact.

To put further stress on this observation, we simulate a drift in the nonuniformity during the time when the camera does not move sufficiently. A slight change is introduced to the FPN at frame 150. Figure 7b shows the PSNR of the two methods where we can clearly see that the drift causes the performance of IRNUC based algorithm to decrease. However, the proposed algorithm quickly adapts its correction parameters to the change, which demonstrates its robustness to such operation conditions.

**Figure 8:**
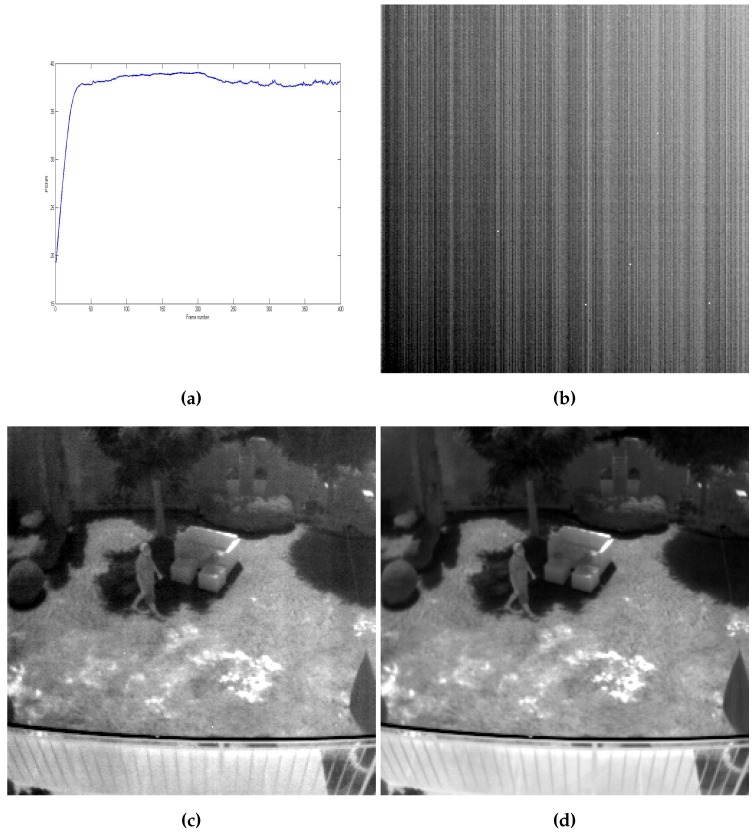
(**a**) PSNR as a function of the frame number of sequence 3; (**b**) real nonuniformity; (**c**) frame 300 corrected using the midway infrared equalization (MIRE) algorithm (PSNR = 32.91); and (**d**) frame 300 corrected using the proposed method (PSNR = 37.86).

Finally, a third sequence was also used to test our method performance in case of real non-uniformity. The real FPN shown in Figure 8b was obtained from a FLIR Tau 320 camera (FLIR Systems, Inc., Wilsonville, OR, USA). It was added to a clear sequence, and the result was corrected using a bias only correction version of the proposed method. Both nonuniform and corrected sequences are represented in video 3 and video 4 respectively. The resulting PSNR is represented in Figure 8a.

Since the real FPN used here is a stripe nonuniformity, we also compared our method to one of the state-of-the-art methods that deal with this kind of nonuniformity, namely the midway infrared equalization (MIRE) algorithm [21]. This approach is a single frame correction that exploits the midway infrared equalization technique to equalize the histogram of each column (or line) using the midway of the histograms of the neighboring columns, hence the name MIRE. Figure 8c,d shows a frame from sequence 3 corrected using the MIRE algorithm and the proposed method, respectively (both images are contrast-enhanced to highlight their differences). The PSNR scored for each method is 32.91 for the MIRE approach and 37.86 for our proposed method. The reason for this difference is, as we can see in Figure 8c, the MIRE method successfully eliminated the stripe pattern, but the high-spatial frequency nonuniformity is still imposed on the image.

### 3.4. Bad Pixel Replacement

**Figure 9:**
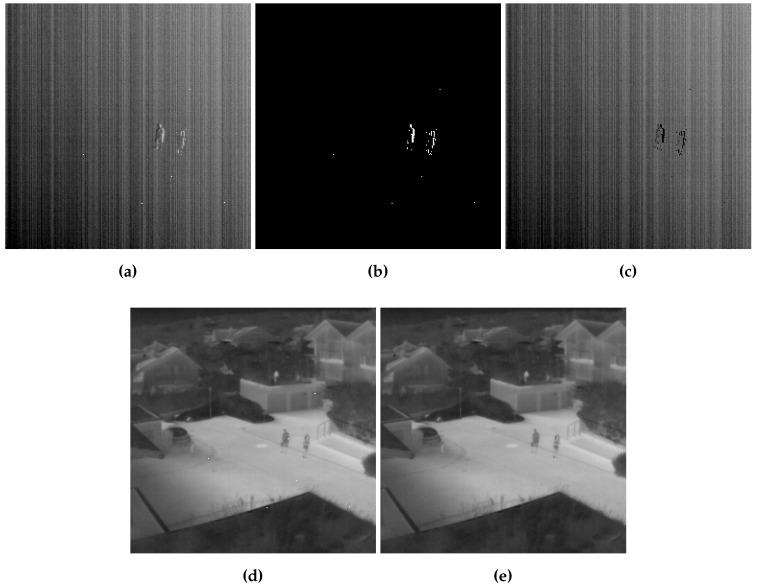
(**a**) registration error; (**b**) abnormal data; (**c**) resulting error used in the correction process; and (**d**) corrected frame without bad pixel replacement; and (**e**) corrected frame with bad pixel replacement.

We have seen in Section 2.3 that during the registration-based correction, the error between two properly registered image frames over the overlapping area may include abnormal data due essentially to local motion. To tackle this problem, we used an exclusion rule to make sure that error used for the correction includes only NU error. However, this rule also excludes abnormal pixels and prevents the correction process from correcting them, which motivates the use of an additional process to replace these pixels as described in Section 2.4. Figure 9 depicts an example showing the effect of the exclusion rule and the bad pixel replacement process. Two frames from sequence 1 corrupted with real nonuniformity were used, and the error after registration is shown in Figure 9a. We can clearly see, in addition to the FPN, the abnormal data in the form of contours of two persons walking with different motion models than the camera, and a few bad pixels. After the use of the exclusion rule, these data are successfully extracted (Figure 9b) and the corresponding pixels will be set to null as shown in Figure 9c. The corrected image frame using the processed error is shown in Figure 9d, as predicted bad pixels are still imposed on the image and degrade its quality. Finally, the replacement of these pixels is conducted and the resulting blind pixels-free image is presented in Figure 9e.

## 4. Discussion

As demonstrated in the previous section through the different experiments, our method outperformed the state-of-the-art methods in terms of rapidity of convergence and accuracy. The balance between the two approaches depending on the level of FPN in the images assured a stable and robust correction and provided a larger set of experimental conditions where the process can work effectively. It is clear that the noise threshold that controls the switching is an important factor to consider in our algorithm in order to work efficiently. One can think of an adaptive threshold to further optimize the outcome of the correction, but, in our work, we demonstrated that using a predetermined fixed value for the threshold would also provide good results and an acceptable level of correction.

We also demonstrated that anti-ghosting measures and bad pixel replacement are essential parts that can be added to the correction process to maintain its stability and enhance the quality of the corrected images. Thus, considering these factors is fundamental in any correction process, especially when used in some applications like detection and surveillance where a bad pixel can engender a false decision.

## 5. Conclusions

We have presented in this work an efficient method to combine two novel scene-based nonuniformity correction techniques for the aim of developing a process that continuously corrects images under any circumstances. The proposed process uses the adaptive NUC method for its high ability to correct and smooth images under a significant level of noise, and uses the IRNUC, which exploits a pure translation motion model between frames, for its effectiveness and preservation of image details, especially under an accurate shift estimates condition.

The proposed method displays great efficiency in terms of the amount of nonuniformity corrected, the convergence speed and the stability of the correction process under different operation conditions. Its comparison with state-of-the-art NUC algorithms showed the gain we can get with a simple combination of classic NUC methods.

In addition, further enhancements were added to the process to deal with problems such as ghosting artifacts and bad pixels. Finally, the overall performance of our method was demonstrated using both simulated and real nonuniformity.

## Supplementary Materials

The following are available online at http://www.mdpi.com/1424-8220/16/11/1890/s1, Video 1: image sequence with simulated nonuniformity, Video 2: correction results through the various approaches (from left to right: adaptive, registration-based and proposed), Video 3: image sequence with real nonuniformity, Video 4: correction results through the various approaches (from left to right: adaptive, registration-based and proposed).

## Abbreviations

The following abbreviations are used in this manuscript:

FPA	Focal Plane Array
IRFPA	Infrared Focal Plane Array
FPN	Fixed Pattern Noise
NU	Nonuniformity
NUC	Nonuniformity Correction
SBNUC	Scene-Based Nonuniformity Correction
LMS	Least Mean Square
IRNUC	Interframe Registration-based Nonuniformity Correction
SBNUC-NN	Scene-Based Nonuniformity Correction Based on Neural Network
DFT	Discrete Fourier Transform
PSNR	Peak Signal-to-Noise Ratio
RMSE	Root Mean Square Error
MIRE	Midway Infrared Equalization

## References

[1] Perry D.L., Dereniak E.L. (1993). Linear theory of nonuniformity correction in infrared staring sensors. Opt. Eng..

[2] Harris J.G. (1999). Nonuniformity correction of infrared image sequences using the constant-statistics constraint. IEEE Trans. Image Process..

[3] Hayat M.M., Torres S.N., Armstrong E., Cain S.C., Yasuda B. (1999). Statistical algorithm for nonuniformity correction in focal-plane arrays. Appl. Opt..

[4] Torres S.N., Hayat M.M. (2003). Kalman filtering for adaptive nonuniformity correction in infrared focal-plane arrays. JOSA A.

[5] Zuo C., Chen Q., Gu G., Sui X., Qian W. (2011). Scene-based nonuniformity correction method using multiscale constant statistics. Opt. Eng..

[6] Hardie R.C., Hayat M.M., Armstrong E., Yasuda B. (2000). Scene-based nonuniformity correction with video sequences and registration. Appl. Opt..

[7] Ratliff B.M., Hayat M.M., Hardie R.C. (2002). An algebraic algorithm for nonuniformity correction in focal-plane arrays. JOSA A.

[8] Ratliff B.M., Hayat M.M., Tyo J.S. (2005). Generalized algebraic scene-based nonuniformity correction algorithm. JOSA A.

[9] Zuo C., Chen Q., Gu G., Sui X. (2011). Scene-based nonuniformity correction algorithm based on interframe registration. JOSA A.

[10] Zuo C., Chen Q., Gu G., Sui X., Ren J. (2012). Improved interframe registration based nonuniformity correction for focal plane arrays. Infrared Phys. Technol..

[11] Zuo C., Zhang Y., Chen Q., Gu G., Qian W., Sui X., Ren J. (2013). A two-frame approach for scene-based nonuniformity correction in array sensors. Infrared Phys. Technol..

[12] Black W.T., Tyo J.S. (2014). Feedback-integrated scene cancellation scene-based nonuniformity correction algorithm. J. Electron. Imaging.

[13] Black W.T., Tyo J.S. (2014). Improving feedback-integrated scene cancellation nonuniformity correction through optimal selection of available camera motion. J. Electron. Imaging.

[14] Scribner D., Sarkady K., Kruer M., Caulfield J., Hunt J., Colbert M., Descour M. Adaptive retina-like preprocessing for imaging detector arrays. Proceedings of the IEEE International Conference on Neural Networks.

[15] Zhang T., Shi Y. (2006). Edge-directed adaptive nonuniformity correction for staring infrared focal plane arrays. Opt. Eng..

[16] Vera E., Torres S. (2005). Fast adaptive nonuniformity correction for infrared focal-plane array detectors. Eurasip J. Appl. Signal Process..

[17] Hardie R.C., Baxley F., Brys B., Hytla P. (2009). Scene-based nonuniformity correction with reduced ghosting using a gated LMS algorithm. Opt. Express.

[18] Guizar-Sicairos M., Thurman S.T., Fienup J.R. (2008). Efficient subpixel image registration algorithms. Opt. Lett..

[19] Lee J.S. (1983). Digital image smoothing and the sigma filter. Comput. Vis. Graph. Image Process..

[20] Portmann J., Lynen S., Chli M., Siegwart R. People Detection and Tracking from Aerial Thermal Views. Proceedings of the IEEE International Conference on Robotics and Automation (ICRA).

[21] Tendero Y., Landeau S., Gilles J. (2012). Non-uniformity Correction of Infrared Images by Midway Equalization. Image Process. Line.

